# The history of the Conference of Research Workers in Animal Diseases (CRWAD) 1920–2014

**DOI:** 10.1017/S1466252315000201

**Published:** 2015-12

**Authors:** Robert P. Ellis, L. Susanne Squires Ellis, Erwin M. Kohler

**Affiliations:** 1 Microbiology, Immunology and Pathology, Colorado State University, Campus Stop 1682, Fort Collins, Colorado 80523-1682, USA; 2 CRWAD Administrative Assistant (Retired), Fort Collins, CO 80526; 3 Professor Emeritus, Food Animal Health Research Program, OARDC, The Ohio State University, Wooster, Oh 44691

**Keywords:** Conference of Research Workers in Animal Diseases (CRWAD), CRWAD history, animal diseases organization, animal diseases research organization, animal diseases research, veterinary diseases research

## Abstract

The following history has been compiled and written by the authors. The historical facts are available from the Conference of Research Workers in Animal Diseases (CRWAD) archives, dating back to letters and summaries written by the founders, and by a few of the Secretary-Treasurers from the early decades through 2014. **The Organization and Purpose**: The CRWAD is a non-profit organization and has been since its origin. The sole purpose of CRWAD is to discuss and disseminate the most current research advances in animal diseases. Graduate students and industry and academic professionals present and discuss the most recent advances on subjects of interest to the CRWAD and of importance to the global livestock and companion animal industries. The oral and poster abstracts of new and unpublished data presented at the meeting sessions are published each year in the CRWAD Proceedings (formerly the CRWAD Abstracts). CRWAD publishes, copyrights, and distributes the Proceedings. The presentations are arranged into the following 10 sections, according to the primary topic of the presentation: Bacterial Pathogenesis, Biosafety and Biosecurity, Companion Animal Epidemiology, Ecology and Management of Foodborne Agents, Epidemiology and Animal Health Economics, Immunology, Pathobiology of Enteric and Foodborne Pathogens, Respiratory Diseases, Vector-Borne and Parasitic Diseases, and Viral Pathogenesis. Prospective members should be actively engaged in animal disease research or research administration. Meeting information and membership applications may be obtained by contacting the Executive Director or by visiting the CRWAD website. Annual abstracts are currently available on-line at the On-line Meeting Planner and Itinerary Builder, with access through the CRWAD website.

## Introduction to the Conference of Research Workers in Animal Diseases (CRWAD)

The Conference of State and National Research Workers in Animal Disease was formally organized on 3 and 4 May 1920, at the Sherman Hotel in Chicago with 14 persons in attendance. At the organizational meeting, a Chairman and Secretary were appointed. There was at least one precursor to the new organization, and that was an Association of Experiment Station Veterinarians which was organized in conjunction with the American Veterinary Medical Association (AVMA) which graciously set aside a number of pages of its Proceedings for use by the newly formed body. There is very little more that was found regarding the Association of Experiment Station Veterinarians, and it is assumed that the organization lasted only for a few years. After the organizing meeting in May 1920, the first meeting of the Conference of State and National Research Workers in Animal Disease occurred on 27 November 1920, at the Sherman Hotel in Chicago. Dr Marion Dorset was the President and Dr R. A. Craig was the Secretary (L. Van Es, 1944, Appendix 1; H. C. H. Kernkamp, 1962, Appendix 2; B. C. Easterday, 1999, Appendix 3).

## Early history

The early history of the Conference has been well described by both Dr L. Van Es's “A Historical Sketch,” November, 1944, Appendix 1; and Dr H. C. H. Kernkamp's “Historical and Reminiscent Review of the Conference of Research Workers in Animal Diseases,” 26–27 November 1962, Appendix 2. Following are excerpts from both papers.

In the words of Dr C. H. C. Kernkamp:

“The idea for organizing a conference and the call for the first meeting in 1920 stems to Dr. R. A. Craig of Purdue University. He was enthusiastically encouraged and actively supported in this venture by the Director of the Purdue Agricultural Experiment Station, Professor C. G. Woodbury. Craig was also urged to go ahead with his idea by his close friend Dr. J. W. Connaway of the University of Missouri.

A bit of reminiscence is ‘sutured-in’ at this point since it was explained to me that Dr. Craig probably got his idea following a dinner in Chicago that was arranged by Dr. L. Van Es of the University of Nebraska. Van Es had taken it upon himself to invite a group of his colleagues to a dinner where, with good wine and cigars, they could carry on what he was want to call ‘more or less scientific shop talk’. He was firm in the belief that much good could come from a ‘bull session’ where scientists could sit down together and ‘pull out the stops’.

Two of these so-called ‘Van Es Dinners’ were held, one in 1918 and one a year later. They were held at the time the United States Live Stock Sanitary Association was holding its meetings. While Van Es and Colleagues were members of the USLSSA and participated in its meetings, he, nevertheless, was of the opinion that it was not the place for free discussions of problems of special interest to research workers. There was present at one or both of the ‘Van Es Dinners’: R. R. Birch, J. W. Connaway, R. A. Craig, Marion Dorset, C. P. Fitch, Ward Giltner, L. W. Goss, Robert Graham, F. B. Hadley, C. M. Haring, Chas. Murray, V. A. Moore, A. F. Schalk, C. H. Stange, and L. Van Es.”

Dr Kernkamp continued with the following:

“Returning to the history of the Conference we find that early in 1920 Dr. Craig corresponded with a number of men in different parts of the country whom he thought might be interested and prospective participants in a meeting to be convened for the purpose of organizing a conference of research workers in animal diseases. He received a sympathetic response which resulted in a meeting in Hotel Sherman in Chicago on May 3 and 4, 1920. At this meeting were R. A. Craig, R. A. Whiting and C. G. Woodbury (Indiana), B. A. Beach and F. B. Hadley (Wisconsin), Ward Giltner and E. T. Hallman (Michigan), M. Dorset (District of Columbia), L. Van Es (Nebraska), A. F. Schalk (North Dakota), C. H. Stange (Iowa), W. W. Dimrock (Kentucky), Robert Graham (Illinois) and C. P. Fitch (Minnesota). Professor Woodbury of the Experiment Station at Purdue was present to assist with the organizing and to get the Conference off on a good start.

In the course of the two-day session various subjects of scientific interest were discussed. Topics on the agenda included: proprietary remedies and bacterins; abortion in cattle and horses; tuberculosis and the tuberculin test; swine plague and swine flu; suipestifer infections in swine; hog cholera virus carriers; swamp fever; and diseases of poultry. The principal item of business was the drafting of a constitution and by-laws to govern the composition and operation of the conference and to set forth its objectives.” (H. C. H. Kernkamp 26–27 November 1962, Appendix 2)

It should be noted that from the very beginning of CRWAD, many of the animal diseases that were studied were zoonotic diseases (tuberculosis and brucellosis, etc.), resulting in a substantial contribution to human health as well as animal health. Thus the One Health concept has been active within CRWAD since the beginning of the organization.

The early history of the CRWAD was stated by Dr L. Van Es as follows:

“The Conference of Research Workers in Animal Diseases was not the first effort to bring about a forum for discussion pertinent to the problems associated with the diseases of livestock. As early as 1897 an Association of Experiment Station Veterinarians was organized in conjunction with the American Veterinary Medical Association, which graciously set aside a number of pages of its Proceedings for use by the newly formed body. For so far as the latter is concerned, available data do not show that it endured for more than a few years. Apparently the larger association then offered a sufficient opportunity for veterinary investigators for an exchange of opinions and inspiration arising from contacts among fellow workers. However, the attempt may serve as an indication that the then prevailing policy of Agricultural Colleges and Experiment Stations to make every farmer his own ‘horse doctor’ was beginning to yield to the growing availability of more or less trained veterinary practitioners. Furthermore, there had gradually developed a more enlightened viewpoint pertaining to the social-economic importance of the diseases of farm livestock.

For some years after Dorset's discovery which resulted in an adequate means of preventing hog cholera, Station Veterinarians and others conferred frequently in order to discuss the more practical phases of the immunization of swine against this scourge. It is quite probable that these meetings more clearly showed the great advantage of a free exchange of opinions and that they may have served as an incentive for the establishment of an organization devoted to the consideration of a wider range of subjects and the problems associated with them.

Since the early years of this century (20th), the functions of Experiment Station veterinarians began to shift increasingly to investigational efforts, in which special appropriations made available by acts of Congress no doubt served as a stimulus. As investigational work by the Experiment Stations as well as of other Government Agencies increased in volume as well as importance, the need of opportunities for contact between individual workers became more and more apparent. Such a need was particularly recognized by Dr. R. A. Craig, of Purdue University, who initiated steps to bring about the organization of the subject of this historical sketch. Available data indicate that Craig had discussed the project with Dr. J. W. Connaway, about two years before the Research Conference was definitely established. Apparently the first steps were taken during the latter part of 1919 when Craig undertook to lay the foundation for an organization. In this he had the active support of his Station Director, C. G. Woodbury, who brought the plan to the attention of his colleagues in the other Stations of the country. It seems that the plan had a favorable reception because in the course of March and April, Craig corresponded with a considerable number of prospective participants of the meeting he proposed to be held.

There was a sympathetic response and a gathering of interested workers, to create a definite organization was assured. It was to erect a structure for which R. A. Craig had already constructed the foundation. Craig and his co-founders as well as others in their willingness to support an organization devoted to research of the diseases of livestock, in a measure reflected trends developing with reference to methods of dealing with such disorders. About the time when Craig conceived a research organization there was already noticeable a drift from curative efforts to preventive ones. Many of the latter required and still require the guidance that can only be procured through well planned research. The Conference of Research Workers in Animal Diseases in North America was established with this in view.”

Dr Van Es explained the desire of those present to keep the organization “simple and that the discussion of research problems be informal.”

Dr Van Es stated:

“The founders of the Research Workers Conference desired to periodically bring together a relatively small group of workers, largely composed of colleagues who knew one another and with whom there could be no hesitancy to present problems arising in investigations, and to seek helpful counsel.

It was agreed that all presentations should be restricted to uncompleted work and finished or published material was frowned upon.

The early meetings were gatherings of friendly colleagues, who engaged in a more or less scientific ‘shop talk’. They agreed or disagreed with one another and returned to their individual problems often better, but never worse because of their participation. Many of the earlier members were lone workers, more or less remote from professional contacts and to them the Conference often yielded inspiration born from the newly acquired assurance, that when beset by vexing difficulties, they were not alone.”

Dr Van Es continued his comments as follows:

“The simple purposes of the organization, as conceived by its Founders, could be readily accomplished as long as the attendance remained small and as long as the information disclosed by the discussions could be accepted by each and all of the participants as confidential. However, it soon became apparent that such an idyllic arrangement may in the long run be difficult to maintain. Even as early as Oct. 8, 1920, Marion Dorset in a letter to Dr. Craig stated; ‘we will have to be careful with this Conference and keep its membership limited, otherwise it will soon become unwieldy and will not serve the purpose for which it is intended.’

Dorset's fears were well grounded, because even during the earlier years of the organization's existence the attendance at its meetings had so increased that the original conception of what it should especially promote had become an impossible realization. Not only had bona fide workers in research become quite numerous, but others, not engaged in investigational work began to flock to the gatherings. There came about an unsolicited attendance largely recruited from the ranks of various state and federal officials and regulatory officers, teachers and others. There can be no doubt that most of such visitors were honestly interested in the discussions by research workers. However, under such circumstances very few of the latter would be inclined to bring before the Conference any problem pertaining to uncompleted work.

This undesirable situation was eventually remedied by amendments of the statutes, by which the qualifications of candidates for membership were defined and which also prescribed the conditions under which non-members may be admitted to the meetings.

The substantial increase of the membership without doubt, brought about certain changes in the more intimate mutual relations between members. Yet, after all, the participation of a greater number of workers must have materially increased the usefulness of the organization. It simply transferred some of the more confidential discussion from the meeting hall to some other place, a phenomenon common to all organizations of a similar character.” (L. Van Es, November, 1944, Appendix 1)

Dr Van Es continued to be a leader in animal disease research and the CRWAD. A tribute to Dr Leunis Van Es (1868–1956) was presented at the 27 November 1956, Business Meeting. The tribute is included as Appendix 4.

Another of the undesirable situations leading to substantial disagreement and uncomfortable situations was the ‘barring’ of non-members, as explained by Dr H. C. H. Kernkamp:

“Over the years a few unpleasantries have crept in on the activities of the Conference. They can be likened to an *abscess*. Abscesses as you know, cause varying degrees of irritation depending on their location, size and stage of development. Moreover, most abscesses are amenable to treatment so that when properly incised and drained, healing follows with a minimum of cicatrisation. One such ‘abscess’ developed at a morning session of the Conference in 1930. Seated toward the rear of the room were several ‘self invited visitors’. From time to time they would carry on a conversation among themselves in audible overtones which tended to distract some of the speakers and disturb some in the audience. As a result the writer was delegated sergeant-at-arms at the noon recess and instructed to tyle the doorway and allow none to pass or repass except they be qualified members. The assignment was not difficult to fill although he was obliged to take a little ribbing and a few withering remarks. But, let it be said, they were made without malice or ill-will.” (H. C. H. Kernkamp 26–27 November 1962, Appendix 2)

## The constitution and By-laws of the CRWAD – debate and contention

The constitution and By-laws have changed several times, but it is clear that the original objectives, established in 1920, remain. Those objectives set forth in the first Constitution are: (1) promote progress in animal disease research; (2) encourage critical review of current research projects; (3) establish mutual understanding and coordination among workers. The latter clause was changed in 1925 to read “Afford opportunity for discussion of results on subjects of interest to the Conference and of importance to the livestock industry.” The above statements have been shortened in the past few years to the summary statement “The sole purpose of CRWAD is to discuss and disseminate the most current research advances in animal diseases. Graduate students and industry and academic professionals present and discuss the most recent advances on subjects of interest to the CRWAD and of importance to the global livestock and companion animal industries.”

The CRWAD meeting has remained in Chicago, except for the years 2001, 2002, and 2005, when it was held in St. Louis, Missouri. There have been only eight hotels in Chicago which have hosted the meeting from 1920 to 2014. There were 12 secretary/treasurers (1920–1991), now designated Executive Directors (1992 – present), during the history of the Conference from 1920 to 2014. Seven of them served before 1950. From 1950 to 2014, CRWAD has been served by only four dedicated individuals, Drs Alfred G. Karlson, Alvin F. Weber, Erwin M. Kohler, and Robert P. Ellis. Dr David Benfield was approved by the Council as the 13th Executive Director at the 2014 annual meeting and began his term as Executive Director 1 January 2015. “While the officers, council members and Conference members are vital to the success of the meetings and the longevity of the Conference, it is clear that these meetings would not happen without the dedication of the Executive Directors.” (B. C. Easterday, 1999, Appendix 3).

Throughout the life of the CRWAD, the Constitution and By-laws have undergone several changes. The fundamental purposes of the CRWAD have remained unchanged, as stated above.

The conference name has been changed three times since the original name was established in 1920, as stated by Dr Kernkamp in his Historical and Reminiscent Review of the conference of Research Workers in Animal Diseases. He referred to these as “rechristenings.” Dr Kernkamp stated those name changes as follows: “CONFERENCE OF STATE AND NATIONAL RESEARCH WORKERS IN ANIMAL DISEASE’ was the original name. It was changed in 1926 to ‘CONFERENCE OF OFFICIAL RESEARCH WORKERS IN ANIMAL DISEASES OF NORTH AMERICA’ and in 1937 to ‘CONFERENCE OF RESEARCH WORKERS IN ANIMAL DISEASES IN NORTH AMERICA.’ The present name, ‘CONFERENCE OF RESEARCH WORKERS IN ANIMAL DISEASES,’ was adopted in 1955.” (H. C. H. Kernkamp 26–27 November 1962, Appendix 2). The last name change reflected on the international interest in CRWAD, and thus opened the meeting to an audience beyond North America and to the discussion of diseases which occur globally. Throughout the years since 1955, the issue of the “name” has been revisited at least once per decade. It has been the determination of the Council that the current name is the best option to date, and that our organization has good national and international recognition due to the 95-year history of CRWAD.

Two very important changes to the By-laws were noted in 1944. Adopted **By-law No. 7** stated:

“Names of members whose annual dues are in arrears for more than 2 years shall automatically be removed from the membership list.”

This stipulation was also listed in the 1939 Action of the Annual Conference. The provisions of Bylaw No. 7 from 1944 are adhered to currently in order to keep the membership roster updated. Throughout the past 40 years of the CRWAD, there have been about 20–25 members dropped each year due to nonpayment of dues for two successive years. **By-law No. 8** stated:

“A member who has been continuously affiliated with the organization for 25 years shall be exempt from the further payment of dues and shall be designated a ‘Life Member’ providing that other prerequisite qualifications for membership are maintained.”

CRWAD has added to the qualifications for Life Membership that the prospective Life Member submits a letter requesting Life Membership, and be fully retired. CRWAD has averaged 105–110 Life Members since 1995. The dedicatee is selected annually from the roster of Life Members.

Those eligible to attend the annual meeting were a point of debate, and at sometimes contention, through the first 40 years of CRWAD. W. H. Feldman was CRWAD Secretary-Treasurer from 1937 to 1948 and President in 1952. Dr Feldman wrote a letter to the CRWAD members on 1 November 1947, wherein he restated the objectives of the summary of the organization as follows: (1) only *new* and *unpublished* data should be presented; (2) the material must be presented in abstract form and *not read*; and (3) presentation shall not exceed a time limit of 10 min. Lantern slides or other illustrative material may be used during the 10 min period of presentation. Dr Feldman went on to reiterate that,

“Membership is confined strictly to research workers employed by state, federal, dominion, or endowed research organizations. When a member severs his connection with any of the above types or organizations, his membership in the Conference automatically terminates. Researchers in commercial organizations, workers in regulatory fields, practitioners, and graduate students are not eligible for membership.”

He went on to elaborate on the types of individuals the Conference desired as members, and indicated that the Conference should not be viewed as a graduate seminar. Admission to the Conference was only extended to members in good standing with membership cards provided by the Secretary, and those non-members specifically invited by the Executive Council (Feldman, 1947, CRWAD archives).

The 30 November 1943, By-law No. 5 which stated,

“Attendance at the meeting of the Conference shall be limited to members of the Conference and to such others as may be invited to attend the meetings by the executive committee. The number ‘eligible’ for membership from any one institution shall not be limited,” was amended 2 December 1947 as follows:

“Attendance to the meetings of this organization shall be limited to duly elected members and to a limited number of guests to be invited by the Council. Invitations shall be restricted to (1) Foreign colleagues who are not undergraduates, (2) distinguished members of the related sciences, and (3) a representative of the Executive offices of the American Veterinary Medical Association. Requests for invitations must be in writing to the Secretary-Treasurer at least two weeks before the date of the annual meeting.”

This was a slight relaxation of the attendance requirements. There were continued extensive discussions both at the Council meetings and at the Business meetings regarding who could be invited as guests. The discussions were between members who wanted to expand and grow the organization and those members who wanted the organization to remain a more closed society. The pros and cons of inviting graduate students were also being discussed in the 1940s.

For the next few years (1948–1954), Constitution By-law No. 5 was included in the meeting “Announcement” as “guidance” and a notice that stated, “A uniformed attendant will be at the door of the meeting and members who attend should have on their persons cards certifying to membership.” This notice continued through 1957 with the word uniformed dropped. The practice of monitoring the meeting room door was addressed a few other times in the minutes and notes which are in the CRWAD archives. One reference (26 November 1951) was from Alfred G. Karlson, Secretary-Treasurer from 1950 to 1964. He noted that the requirement to limit the attendance to members and invited guests provided “a great deal of embarrassment because it is necessary to abide by the constitution which definitely limits the number of guests and also defines the requirements for membership.” (A. G. Karlson 1951 CRWAD Archives) Name tags were initiated at the 1952 meeting to assist the Secretary-Treasurer in determining who was permitted to attend the meeting.

Article II of the Constitution was amended 2 December 1947. The words, “employed by the United States Government or by the national, state or provincial government in North America or by endowed institutions” were deleted. The word “society” was changed to “conference” and the words “except graduate students” were deleted. This allowed for the continuation of membership if a member went back to school.

On 27 November 1949, the Report of the Acting Secretary-Treasurer to the Council, Alfred G. Karlson stated,

“The number of requests from members to invite non-members to attend the annual meeting is decreasing. Apparently most of the members are now aware of the Constitutional requirement that only the Council is permitted to invite guests. In keeping with the established practice, however, the acting secretary has issued invitations to non-members whose papers have been accepted for presentation at the meeting. In each instance the non-member was the first author of the paper and associated authors were members.”

The 28 November 1949, Business Meeting Minutes state,

“In response to the request of the President G. S. Harshfield there was some discussion from the floor regarding the admission of guests, certain non-members especially graduate students. It was pointed out by one member that most of the research is being done by graduate students and that they will be the leaders of the future. To deny them the opportunity of attending the Conference meetings is undemocratic. Following this there was some discussion regarding the institutions that would bring graduate students.”. . . . . . “A number of statements were made supporting the desire to admit graduate students as guests. There were also some statements to the contrary. One member said that to admit graduate students would only increase the attendance which was already too large and that graduate students would not contribute to the program. The secretary was asked to read the portion of the Constitution pertaining to amending the Constitution. The President closed the discussion by saying that it was the privilege of any member to submit in writing any change that was deemed advisable in the Constitution.” (Business Meeting Minutes, 28 November 1949, CRWAD archives)

This discussion opened the door for graduate students to be full participants in the annual meeting. It took 49 years, but in 1998 the Council recommended and the members approved at the annual business meeting the addition of a Student Member category to be fully implemented in 1999. The students would not vote, but would be included in the Call for Abstracts, the Newsletter, and all other communications that go to the CRWAD members. Student membership is for 1 year, renewed by conference attendance. The reasons were that the students had for decades been the largest group of presenters at the annual meeting, and including them as student members would encourage them to become active members after they were established in their research careers. In the 16 years since that acknowledgement of the students’ contributions to CRWAD, there have been many who have gone on to become active members.

There was another significant change in the Constitution regarding membership in 2004. Prior to the change in membership requirements, Article III of the CRWAD Constitution read as follows:

“Prospective members shall be actively engaged in research or research administration. They shall present to the Council the name of the research project(s) on which they are working, a list of their research publications, **and 3 reprints of work published as senior author in refereed journals within the past five years.** Case reports, abstracts, general reviews or popular articles not acceptable. Research administrators with evidence of earlier accomplishment will be eligible. Applicants for membership must submit to the Executive Director an application **supported by a letter from each of two members of the CRWAD. (Emphasis added by R. P. Ellis)** The application must be acted upon favorably by the Council and approved by the Conference in regular session.”

Article III of the CRWAD Constitution as amended 16 November 2004:

“Prospective members shall be actively engaged in research or research administration. They shall present to the Council the name of the research project(s) on which they are working and a list of their research publications in refereed journals. Case reports, abstracts, general reviews, or popular articles are not acceptable. Research administrators with evidence of earlier accomplishment will be eligible. Applicants for membership must submit an application and CV to the Executive Director of the CRWAD. The application must be acted upon favorably by the Council and approved by the Conference in regular session.”

For the past 10 years this Amendment regarding membership qualifications has been in effect, there has been no measurable change in the overall quality of the new members granted membership in the CRWAD.

According to the Constitution, the first dues, $1.00 per member, were levied in 1926 and remained static through 1951. As inflation began to take a toll, the dues were subsequently increased. In 1970 the dues were $3 and $5 in 1984. It was critical that the dues/registration be increased enough to erase the deficit experienced in 1990, so the Council approved an increase at the 1990 meeting, which went into effect in 1991, of $15 for members, $25 for non-member guests, and $10 for students. Over the years as the services of the CRWAD have increased, and as we added an Administrative Assistant, L. Susanne (Suzy) Squires Ellis, the dues and registration were increased to cover the increased costs. In 2005, the Council approved another change in dues/registration. CRWAD members were assessed $70 membership annual dues and meeting registration was $40 for students and post docs, $60 for members, and $175 for non-member guests. Membership dues remained $70 through 2014, with registration for students at $70, $90 for post docs, $120 for members, and $325 for nonmember guests.

To conclude the section on the Constitution and By-laws of the CRWAD, it should be noted again that from the origin of CRWAD, it was a not-for-profit organization. CRWAD had applied and was granted a Business Identification Number during the time that Dr Erwin Kohler was the Secretary-Treasurer. However, since CRWAD had never taken in receipts valued annually at more than $25,000, it was not required to file tax returns. CRWAD exceeded the ceiling in 1998. CRWAD applied for and was granted the IRS 501 (c) (3) not-for-profit Corporation designations by the Department of the Treasury on 18 February 1999. Since CRWAD Executive Director was located at Colorado State University in Fort Collins, Colorado, CRWAD became an entity transacting business in Colorado 1 January 2004, and became a Colorado not-for-profit Corporation 26 October 2007.

## The CRWAD Council

At the beginning of the CRWAD, there was no Council. From 1920 until 1939, the only officers were the President and the Secretary-Treasurer. The CRWAD annual meeting was essentially a closed meeting with up to two representatives from each of the approved entities, so there was no need for further representation. The Vice President was added in 1939. An “Executive Council Member” was first mentioned in the 1947 Council minutes. In 1948 members of the Council consisted of the President, Vice President, Secretary-Treasurer, and four Council Members. The Council Members moved up each year to culminate with the presidency and this Council structure has continued through 2014, the date this history covers.

Throughout the first 65 years of the CRWAD, it truly was an “old boys club” with mostly men in attendance during the early years, and only men on the Council until 1985. Dr Lynette Corbeil was the first woman elected to the Council, and she ascended through the Council to the role of Vice President in 1989, and President in 1990. To date, all dedicatees have been men, and that will most certainly also change very soon.

During the years of World War II, 1940–1945, the Council debated whether to continue to meet or to suspend meetings for the duration of the war. Council justified continuing to meet, and according to Secretary-Treasurer William H. Feldman, inserted the following statement into the announcement (which became later the Call for Abstracts) of the Annual Conference of Research Workers in Animal Diseases in North America,

“In deciding whether or not to attend this meeting, it might be well to remember that the members of this organization are charged with the responsibility of keeping animal diseases at a minimum. The function of research in providing protection for our vital domestic animal resources was never more necessary than now. New facts provide new weapons in the never ending conflict with disease processes.” During the 1941 meeting, on December 1, 1941, the Council approved, “Canadian members were exempted from paying annual dues for the duration of the War.”

Under the current By-laws (approved at the 2013 Business Meeting), the Council consists of the six members (President, Vice President, and the four Council members), plus the Executive Director as an *ex officio* member. The members of the nominating committee are appointed by the current President, and they are given some guidance as to how to nominate the next Council member, the one who will replace the President. Usually the new Council member is chosen from within the discipline or sub-discipline that the President represented. In this way there is an understanding that the Council will be somewhat representative of the membership of the CRWAD. This premise of representation is also true of the section leaders and was acknowledged in the 1970 Council Minutes which encouraged section leaders to seek successors from a different institution and where applicable with a different specialty orientation.

The Council is charged with specific duties, including designating an annual dedicatee and auditing the CRWAD finances. The Vice President has been officially in charge of the dedicatee nominations and voting since 2008. The dedicatee nomination form has been on-line at the CRWAD web pages since 2010, in an effort to engage the members in nominating and supporting the nomination of the dedicatee. After the list of eligible dedicatee nominees has been determined, the Vice President sends out the ballots to the persons charged with electing the dedicatee. The current seven Council members plus the most immediate four past Presidents (the list of Presidents is Table 1) are the 11 persons who cast ballots for determining the dedicatee. The dedicatee tradition was initiated by CRWAD President Bernard C. Easterday and began in 1974, when Dr W. R. Hinshaw was named the first dedicatee. The list of dedicatees from 1974 to 2014 is Table 2. Over the 41 years of the awarding of the dedicatee, the dedicatees have been the pillars of not only the CRWAD, but also of the animal disease research community.

**Table 1.** CRWAD Presidents, Secretary-Treasurers, meeting dates and meeting Hotels. Compiled from archive records, notes and minutes by L. Susanne Squires Ellis and Robert P. Ellis

Meeting date	Hotel name	President	Secretary-Treasurer	Meeting
3–4 May 1920	Sherman	L. Van Es	R. A. Craig	Organize
27 November 1920	Sherman	Marion Dorset	R. A. Craig	1st
26 November 1921	Sherman	Marion Dorset	R. A. Whiting	2nd
5 April 1905	Sherman	C. H. Stange or L. Van Es	R. A. Whiting	3rd
6 April 1905	Sherman	C. H. Stange	R. A. Whiting	4th
7 April 1905	Sherman	L. Van Es	R. A. Whiting	5th
8 April 1905	Sherman	M. Dorset	L. W. Goss	6th
30 November 1926	Sherman	C. P. Fitch	A. F. Schalk or L. W. Goss	7th
29 November 1927	Sherman	C. P. Fitch	A. F. Schalk	8th
4 December 1928	Sherman	C. P. Fitch	A. F. Schalk	9th
3 December 1929	Sherman	C. P. Fitch	A. F. Schalk	10th
2 December 1930	Sherman	Ward Giltner	A. F. Schalk	11th
1 December 1931	Sherman	Ward Giltner	A. F. Schalk	12th
29 November 1932	Sherman	Ward Giltner	A. F. Schalk	13th
5 December 1933	Sherman	R. L. Rettger	L. M. Pickens & A. F. Schalk	14th
4 December 1934	Sherman	E. A. Watson	A. F. Schalk	15th
3 December 1935	Sherman	W. E. Cotton	A. F. Schalk	16th
1 December 1936	Sherman	L. Enos Day	A. F. Schalk	17th
30 November 1937	Sherman	A. F. Schalk	W. H. Feldman	18th
29 November 1938	Sherman	W. A. Hagan	W. H. Feldman	19th
5 December 1939	Sherman	C. Murray	Wm. H. Feldman	20th
3 December 1940	Palmer House	I. E. Newsom	W. H. Feldman	21st
2 December 1941	Palmer House	Hadleigh Marsh	W. H. Feldman	22nd
1 December 1942	Palmer House	E. T. Hallman	W. H. Feldman	23rd
30 November 1943	Palmer House	R. R. Birch	W. H. Feldman	24th
5 December 1944	Palmer House	W. H. Schoening	W. H. Feldman	25th
4 December 1945	Palmer House	General Raymond A. Kelser	W. H. Feldman	26th
3 December 1946	Palmer House	H. E. Biester	W. H. Feldman	27th
2 December 1947	Palmer House	E. L. Stubbs	W. H. Feldman	28th
29 November 1948	Palmer House	H. C. H. Kernkamp	W. H. Feldman	29th
28 November 1949	Palmer House	G. S. Harshfield	A. G. Karlson (acting S-T)	30th
27 November 1950	Palmer House	C. A. Brandly	Alfred G. Karlson	31st
26 November 1951	Palmer House	W. L. Boyd	Alfred G. Karlson	32nd
1–2 December 1952	Palmer House	Wm. H. Feldman	Alfred G. Karlson	33rd
30 November–1 December 1953	Palmer House	F. W. Schofield	Alfred G. Karlson	34th
29–30 November 1954	Palmer House	H. J. Stafseth	Alfred G. Karlson	35th
28–29 November 1955	Palmer House	E. P. Johnson	Alfred G. Karlson	36th
26–27 November 1956	Hotel Maryland	B. T. Simms	Alfred G. Karlson	37th
2–3 December 1957	Hotel Maryland	S. H. McNutt	Alfred G. Karlson	38th
1958	Hamilton	Carl Olson, Jr	Alfred G. Karlson	39th
1959	Hamilton	R. D. Turk	Alfred G. Karlson	40th
1960	Hamilton	Cecil Elder	Alfred G. Karlson	41st
1961	Hamilton	Hugh S. Cameron	Alfred G. Karlson	42nd
1962	Hamilton	Henry Van Roekel	Alfred G. Karlson	43rd
2–3 December 1963	Hamilton	Harry G. Downie	Alfred G. Karlson	44th
30 November–1 December 1964	Midland	Chester Manthei	Alfred G. Karlson	45th
29–30 November 1965	Midland	C. K. Whitehair	Alvin F. Weber	46th
28–29 November 1966	Midland	Wayne Binns	Alvin F. Weber	47th
27–28 November 1967	Midland	W. L. Sippel	Alvin F. Weber	48th
1–2 December 1968	Midland	H. E. Moses	Alvin F. Weber	49th
1–2 December 1969	Midland	Donald Ingram	Alvin F. Weber	50th
30 November–1 December 1970	Midland	R. W. Dougherty	Erwin M. Kohler	51st
29–30 November 1971	Midland	G. R. Spencer	Erwin M. Kohler	52nd
27–28 November 1972	Midland	A. F. Sellers	Erwin M. Kohler	53rd
26–27 November 1973	La Salle	A. L. Kleckner	Erwin M. Kohler	54th
2–3 December 1974	La Salle	Bernard C. Easterday	Erwin M. Kohler	55th
1–2 December 1975	La Salle	L. E. McDonald	Erwin M. Kohler	56th
29–30 November 1976	Pick Congress	J. E. Breazile	Erwin M. Kohler	57th
28–29 November 1977	Pick Congress	D. D. Goetsch	Erwin M. Kohler	58th
27–28 November 1978	Pick Congress	R. B. Wescott	Erwin M. Kohler	59th
26–27 November 1979	Pick Congress	N. F. Cheville	Erwin M. Kohler	60th
10–11 November 1980	Pick Congress	J. B. Derbyshire	Erwin M. Kohler	61st
9–10 November 1981	Americana Congress	E. O. Haelterman	Erwin M. Kohler	62nd
8–9 November 1982	Americana Congress	David Van Sickle	Erwin M. Kohler	63rd
14–15 November 1983	Americana Congress	Harold E. Dziuk	Erwin M. Kohler	64th
12–13 November 1984	Americana Congress	B. I. Osburn	Erwin M. Kohler	65th
11–12 November 1985	Americana Congress	S. A. Ewing	Erwin M. Kohler	66th
17–18 November 1986	Americana Congress	Roland Dommert	Erwin M. Kohler	67th
16–17 November 1987	Americana Congress	William Mengeling	Erwin M. Kohler	68th
14–15 November 1988	The Congress	Alvin F. Weber	Robert P. Ellis	69th
6–7 November 1989	The Congress	William Wagner	Robert P. Ellis	70th
5–6 November 1990	The Congress	Lynette Corbeil	Robert P. Ellis	71st
11–12 November 1991	The Congress	Robert M. Corwin	Robert P. Ellis	72nd
			Executive Director	
9–10 November 1992	The Congress	Richard F. Ross	Robert P. Ellis	73rd
Meeting date	Hotel name	President	Executive Director	Meeting
8–9 November 1993	The Congress	Lawrence H. Arp	Robert P. Ellis	74th
14–15 November 1994	Ramada Congress	Ronald D. Schultz	Robert P. Ellis	75th
13–14 November 1995	Ramada Congress	Bradford B. Smith	Robert P. Ellis	76th
11–12 November 1996	Ramada Congress	Patricia E. Shewen	Robert P. Ellis	77th
10–11 November 1997	Ramada Congress	Bert E. Stromberg	Robert P. Ellis	78th
8–10 November 1998	The Congress	Donald G. Simmons	Robert P. Ellis	79th
7–9 November 1999	The Congress	M. D. Salman	Robert P. Ellis	80th
12–14 November 2000	The Congress	Leon N. D. Potgieter	Robert P. Ellis	81st
11–13 November 2001	Millennium	Linda J. Saif	Robert P. Ellis	82nd
10–12 November 2002	Millennium	Franklin A. Ahrens	Robert P. Ellis	83rd
9–11 November 2003	Congress Plaza	Katherine M. Kocan	Robert P. Ellis	84th
9–11 November 2004	Congress Plaza	Janet MacInnes	Robert P. Ellis	85th
4–6 November 2005	Sheraton Westport	Ian Gardner	Robert P. Ellis	86th
3–5 December 2006	Marriott Downtown	Prem S. Paul	Robert P. Ellis	87th
2–4 December 2007	Marriott Downtown	Lynn A. Joens	Robert P. Ellis	88th
7–9 December 2008	Marriott Downtown	Richard E. Isaacson	Robert P. Ellis	89th
6–8 December 2009	Marriott Downtown	R. William Stich	Robert P. Ellis	90th
5–7 December 2010	Marriott Downtown	Eileen L. Thacker	Robert P. Ellis	91st
4–6 December 2011	Marriott Downtown	Laura L. Hungerford	Robert P. Ellis	92nd
2–4 December 2012	Marriott Downtown	Donald L. Reynolds	Robert P. Ellis	93rd
8–10 December 2013	Marriott Downtown	Rodney A. Moxley	Robert P. Ellis	94th
7–9 December 2014	Marriott Downtown	David A. Benfield	Robert P. Ellis	95th

**Table 2.** Conference of research workers in animal diseases (CRWAD) Dedicatees 1974–2014

W. R. Hinshaw	1974	Edward H. Bohl	1995
S. H. McNutt	1975	Lyle E. Hanson	1996
H. C. H. Kernkamp	1976	Gordon R. Carter	1997
R. W. Dougherty	1977	J. Brian Derbyshire	1998
C. H. Brandley	1978	Bernard C. Easterday	1999
S. F. Scheidy	1979	Leroy Coggins	2000
A. G. Karlson	1980	David P. Anderson	2001
I. A. Merchant	1981	Johannes Storz	2002
L. C. Ferguson	1982	Alexander J. Winter	2003
Fred Maurer	1983	Harley W. Moon	2004
Carl Olson, Jr	1984	William L. Mengeling	2005
Charles Cunningham	1985	Leland E. Carmichael	2006
Ben S. Pomeroy	1986	Richard F. Ross	2007
Norman Levine	1987	Sidney A. Ewing	2008
Earl Splitter	1988	Norman F. Cheville	2009
Marvin J. Twiehaus	1989	Samuel K. Maheswaran	2010
R. Allen Packer	1990	Donald G. Simmons	2011
Donald A. Barnum	1991	William C. Wagner	2012
Alvin F. Weber	1992	Fredric W. Scott	2013
E. O. Haelterman	1993	Donald C. Robertson	2014
Erwin M. Kohler	1994		

The two junior Council members are the auditing committee. After many years of “twisting the arms” of members to serve as the auditing committee, it was suggested by the Administrative Assistant (Suzy Squires) that the two junior members of the Council serve annually as the auditing committee. This has proven to be a very efficient and timely manner for conducting the annual audit of the CRWAD finances since it was initiated in 2009. In addition to the Council audit, the CRWAD accountants perform an in-depth audit every 3–4 years.

Dr Erwin M. Kohler commented that he could not exactly remember how he became the “victim” (the next Secretary-Treasurer). Dr Kohler first attended CRWAD in 1965. He thought possibly that Dr Fergusen, who had become the Department Head of the Ohio Agricultural Research and Development Center (OARDC), asked if he was interested. Dr Kohler stated, “I don't know why I said yes, but I believed in the way the meeting served a real purpose. I did enjoy most of it and all the cooperative people I worked with. The CRWAD President, Dr Bob Daughtery made a special visit to Wooster to find out if I was ‘up to’ the task. He had supper at our house and my 11-year-old son spilled a glass of ice water on him.’ Dr Kohler passed that test and went on to serve the CRWAD with distinction for 18 years as Secretary-Treasurer.”

Secretary-Treasurer Dr Erwin M. (Erv) Kohler asked Dr Robert P. (Bob) Ellis in mid-summer 1987 if he would consider being the next Secretary-Treasurer, which was the office Erv held for 17 years. Bob was very familiar with the CRWAD. He attended CRWAD for the first time as a graduate student from Purdue in 1967 and presented his first paper at CRWAD in 1968. (Thus began Bob's 47-year history with CRWAD, 1967–2014). During this time Bob has only missed two meetings, 1971 when he had recently moved from graduate student status at Purdue University to become an Assistant Professor at South Dakota State University, and one in the mid-1980s when he had been in Peru conducting animal disease research. Bob's reply to Erv was he would “think about it,” and then a week or so later told him that he would accept the role. The Council approved the retirement of Dr Kohler and the new role for Dr Ellis. Erv included Bob in some of the planning for the 1987 meeting by phone calls. As stated above, Bob had been a presenter at CRWAD since 1968, and had been a section leader, so he knew how to notify the section leaders, and how to plan and balance the final schedule. Bob stated that “Dr Kohler coached him on how to balance the sections, choose the section leaders, and communicate with the section leaders and presenters.” The complete transition occurred after the 1987 meeting, and the Secretary-Treasurer position was Bob's. Erv gave Bob the membership list in a notebook on 3 × 5 index cards, and transferred the funds. That finalized the transfer.

In the fall of 1988 (Bob's first year “flying solo”), Monday before abstracts were due, he had received 40 (all hard copies which were mailed, which was the only way to send them at that time), about 10% of what was expected for the meeting. Bob called Erv for advice, and his advice was to be patient, the abstracts would be there by the deadline. Bob left that afternoon on a field trip to SW Colorado, working on sheep ranches and had no contact with the home office. He came back to the office Friday afternoon, had two boxes full of abstracts, and sure enough, a total of about 400 were received just before the deadline. Even as the submission format for the abstracts has changed, from hard copies mailed in, to electronic attached to emails, to the current on-line Abstract submission and Information System (OASIS), it is still the norm that at least 90% of the abstracts are received in the last few hours prior to the deadline. Bob has always been grateful for Erv's advice, on this and many other topics.

The Council presented an honorarium to Dr Erwin Kohler for his 18 years of service as the Secretary-Treasurer. Dr Kohler and Mrs Gelsey Kohler received two round trip airline tickets to attend the International Veterinary Epidemiology and Preventative Medicine Meeting, in Stockholm, Sweden. Dr Kohler stated that it was a great meeting and included scientists from the Soviet Union.

In 1990, after our meeting, CRWAD did not have enough funds to cover our expenses. The Secretary-Treasurer, Bob Ellis, personally opened and secured a line of credit ($4500) so we could operate until we were able to raise meeting registration and build enough funds to cover our debts. CRWAD generated enough funds over the next few years to fully pay the line of credit, and has not needed to borrow again to stay solvent. It has been an annual struggle, but we have been able to build a reserve so that we should never be in a debt position again.

Operating capital has always been a concern for the Council. The 1965 CRWAD Secretary-Treasurer Dr Alvin Weber expressed this financial concern in his comments included when he submitted a completed survey form from the Independent Nonprofit Institutions Studies Group of the National Science Foundation. He filled it out for the fiscal year ending November 1964, and submitted it on 14 September 1965, 50 years ago from the date of the compilation of this history. There were no full-time or part-time employees, total number of members was 350, and total expenditures were $350, with a note stating “just enough for stationery and stamps to run the organization.” He added that “some day we may be large enough to justify the postage spent on this survey!” (CRWAD Archives).

Through experience with other organizations, and advice from our accountants in Fort Collins, CO, Bob learned that an organization such as CRWAD should have in reserve at least the equivalent of one year's operating funds. The Council agreed and in 1992 minutes stated, “It was suggested that we accrue at least 1 year's operating expenses to be held in reserve for the event of a cancellation of the meeting, natural disasters, etc.” Throughout the years that goal was reached, and at the end of his term as Executive Director, the reserve funds for CRWAD were at least enough to carry the organization for 1 year in case a catastrophic event occurred that prevented holding the annual meeting. Also in 1992, the Council directed the Executive Director to “set up a budget and purchase software to assist in financial accounting.” This was accomplished at first by carefully projecting the operating and meeting expenses and receipts for the coming year, and for a few years beyond so that we could anticipate what would be needed to continue to operate in the black, and by gradually building at least 1 year of reserve funds. The purchase of the Quicken Financial Program in 1997 allowed us to keep electronic records of receipts and disbursals and to build more accurate budgets. The annual meeting is the event that generates the most income for CRWAD. In order to further protect CRWAD from natural or other disasters which could affect our annual meeting, Event Cancellation Insurance was purchased in 1999 for the first time, and has been purchased for every year since.

The Council observed that the duties and the time required of the Executive Director were increasing. At the 1993 Council meeting, the Council determined to pay the Executive Director an honorarium as partial compensation for the Executive Director's duties, if there was enough money in the CRWAD account. An honorarium was paid to the Executive Director continuously from 1993 to 2014.

In 1993, Linda Susanne (Suzy) Squires Ellis (Bob's spouse) began doing clerical and data entry work for the CRWAD, and worked at the on-site meeting registration. She continued to work as hourly contract labor from 1993 through 2007. In 2008 Suzy became the first and only employee for the organization until she resigned on 31 December 2014. Though the job title was Administrative Assistant, Suzy actively worked in all levels of the CRWAD business with Bob Ellis as the responsibilities of the Executive Director and the Administrative Assistant began to mount. Suzy developed and posted the CRWAD website in 1997. She managed the website until she transferred it to the new Executive Director in June 2015. The website became an important method of communicating with the membership beginning in 1998. She developed our databases, and has done all our accounting for many years. As the only employee, Suzy did the monthly corporate business work, compiled the annual Program and Proceedings, and organized the annual satellite meetings, keynotes, and exhibitors. In Bob's words and in the opinion of many who attend the CRWAD meetings, “Suzy is the one who gets the Proceedings and Program to the publisher and deserves much of the credit for how smoothly the entire annual meeting runs.” In addition, she assisted with hotel contracts and meeting arrangements. She kept complete records of the finances and membership, and handled most of the correspondence and other day-to-day activities. Bob states, “CRWAD and its meeting were frequent, almost daily, topics of discussion during breakfast and supper. That way we could keep each other informed as to what had transpired during the day and what needed to be done during the next few days. From August through December, the daily activities built for each of us until the meeting had concluded and the hotel was paid. Then after we got home, Suzy had a lot of year-end accounting and other correspondence before she could ‘slow down.’ After resigning the employee position, she continued to work through 2015 for the organization as contract labor, concluding the transition to the new Executive Director, Dr David A. Benfield.”

At the end of 2014, Bob Ellis had completed 27 years as the Secretary-Treasurer (1988–1991) and then Executive Director (1992–2014) of CRWAD. Peggy and Bob Lewis assisted with registration for most of those years. For most of the years that Bob Ellis was Executive Director, it was Suzy Squires Ellis, Peggy Lewis, and Bob Lewis at the registration table welcoming members, students, and guests to the annual meeting. The attendees always remarked that it was nice to see the same friendly faces when they came to the CRWAD meeting. Prior to the availability of pre-registration, which began in 1998, the line for registrations would always be at least 20 people long all afternoon on Sunday before meetings began on Monday morning. Though it moved forward at a steady pace, it never seemed to get much shorter until the end of the afternoon. There was a lot of friendly visiting that occurred in the registration line, and very few ever complained that the registration process took too long. The registration line, though possibly frustrating for some, afforded time to reunite with colleagues and friends.

## The annual meeting

The annual meeting centers on the presentation of current and largely unpublished research in a wide array of animal disease topics. What began as a small invitation-only meeting (attended by about 20 men) has evolved into a large meeting of international scientists presenting their research on diseases of global importance.

It is written in many of the memoirs of that first meeting that it grew from the Van Es dinners which were held during the International Livestock Exposition in Chicago. The International Livestock Exposition was held during Thanksgiving week from 1899 to 1973. For many years, until 1980, the CRWAD annual meeting was the Monday after Thanksgiving. Later when the meeting was changed to a 2 day meeting (1952), it was Monday and Tuesday after Thanksgiving. From 1980 to 2005, the meeting was held the second weekend of November. This move to earlier in November was an attempt to avoid inclement weather for the meeting. In large part it seemed that even though there was the occasional very cold and stormy second weekend in November, the move on average was for the better.

A more “extreme” move, which occurred in 2001, 2002, and 2005, was that the meeting was held in St. Louis. These were the only 3 years in the history of the CRWAD that the annual meeting was not held in Chicago. Dr Ellis presented the idea of meeting in another mid-west city to the Council at the 1999 meeting. The reason stated was simply economics. Of several cities considered, St. Louis was the city chosen due to its choices of meeting hotels, and the ease of flying to St. Louis. CRWAD obtained tax-exempt status from the state of Missouri, but the state of Illinois would not grant tax-exempt status, even after several attempts. In addition, the overall meeting cost in St. Louis was about 15% less than in Chicago. Overall savings were about 25% over the cost of the meeting in Chicago. In addition the sleeping room rates were much lower. The idea was approved by the Council, and the members were canvassed. The majority of the members agreed, so the 2001 and 2002 meetings were at the Millennium Hotel in St. Louis. The members were again canvassed and by a large majority wanted to meet only in Chicago. The reasons given were essentially tradition and familiarity with the city. At the 2002 meeting in Chicago, Dr Ellis and his wife Suzy Squires contacted several hotels in Chicago to explore other venues which would be able to host our meeting. Many hotels do not have the meeting breakout space for an organization such as CRWAD, which requires up to eight meeting rooms on Monday and Tuesday, plus several other options for the receptions, tabletop exhibits, and satellite meetings. In addition, the members had stated clearly that they would rather meet in a better hotel than the Congress, which had gradually become more in need of repairs each year we met there. In fact, the last few times we met at the Congress, there were obvious signs of neglect, including a break in a water pipe which caused a steady drip in the meeting room. The drip happened to be in line with the projector, so all through the sessions in that room there were flashes of the drips passing through the projector light. Throughout the years there were many stories of the “ambiance” of the Congress, including no hot water in some rooms, no towels in some rooms for several days, mice playing on the bed while people were in the bed, and surely there were more such occurrences that were not reported.

The Ellises were able to contract the Marriott at 540 North Michigan Avenue for the meeting in 2006 and a few following years. The Marriott is a high quality hotel, both in terms of meeting space and service from the hotel staff. As of 2014, CRWAD has annual contracts with the Marriott through 2018. The sleeping room rates were reasonable for Chicago in that quality of hotel only if we would meet a week after Thanksgiving. The hotel would not have room for CRWAD before Thanksgiving due to city-wide conventions, and our attendees would not have paid the sleeping room rates that were available for earlier dates. So, we were back to the higher probability of weather problems by meeting later in the year. The first year of our contract with the Marriott, 2006, CRWAD met Sunday to Tuesday, 3 to 5 December, and satellite meetings began Friday, 1 December. On the Thursday before the meeting was to start on Sunday, it began to snow and blow, with a blizzard in full force throughout the Midwestern states of the USA. The airports and highways were closed throughout the central Midwest, so there was no way to get to Chicago. Bob stated, “From our room on the 45th floor, we could not see the streets below us, nor the buildings a half-block away from us. The blizzard did blow itself out pretty quickly, and most people were able to get to the meeting by Sunday.” A few of those attending Friday and Saturday satellite meetings were a day late, and the satellite meetings were either moved a day later or cancelled. Bob further stated, “I am sure there had been other such storms that affected the CRWAD meetings, but since I began attending in 1967, that was the worst I saw. In addition, it was my responsibility to ensure that we had our annual meeting, but this ‘act of God’ was certainly testing my confidence in the scheduling of our meeting in early December.”

There were only 10 oral presentations at the 1921 meeting. That number increased to 22 at the 1930 meeting, and held relatively stable at 16–20 through 1944. The number of presentations began to increase in the 1950s with 53 presentations in 1957, then 156 in 1970, 302 in 1980, 350 oral and 88 posters in 1990, and 171 oral, 86 posters and 8 Keynotes in 2014.

The Secretary-Treasurer was approved in 1939 to issue membership cards to those in good standing to certify CRWAD membership. This was to ensure that only members were allowed to attend the meeting.

The attendance at the annual meeting has varied from around 14 to 20 in the early years to the peak years in the mid-1980s to the mid-1990s of 600–700. The highest attendance was 740 at the 1990 meeting. The average from 1990 to 1999 was 660. Largely due to the curtailing of funding for animal disease research, the attendance from 2010 to 2014 averaged 433. The number of attendees is not a good measure of the scientific importance of the annual CRWAD meeting. The importance is gauged more by the quality of the abstracts presented, and the quality has remained superb.

As the meeting changed to include more participants and more presentations, the organizers saw the need to review the abstracts and select those which were the best of the submissions for presentation. The first mention of “refereed reports” was for the 1930 meeting. Throughout the ensuing decades, there has been a measure of refereeing of the submitted abstracts. That is currently done by the section leaders and co-leaders.

For many years, there have been mini-symposiums in the different sections. The first of these mini-symposiums mentioned was on Bovine Mastitis in 1945. The mini-symposiums present the opportunity to focus on a particular topic or sub-topic within the section. Throughout the past few decades, there have been many symposiums and mini-symposiums held either as satellite meetings prior to or after the annual meeting, or incorporated within a particular section. The section leaders and co-leaders have used these mini-symposiums to generate interest within their particular sections, and in some instances to re-generate enthusiasm for their particular section.

The addition of the Keynote Speakers was started by the Immunology Section. At the 1997 meeting, the scheduled 1:30 pm abstract in the Immunology section had been cancelled and the section filled the 15 min with a lecture by the recently-selected Distinguished Veterinary Immunologist. The Distinguished Veterinary Immunologist is selected annually by the American Association of Veterinary Immunologists. Beginning in 1998 it was a tradition approved by the Council that the Distinguished Veterinary Immunologist was given the first 30 min after lunch on Monday. That time was extended to 45 min a few years later. Dr Ellis suggested that the Keynote concept was working very well for the Immunology section, and should be extended to all sections. He suggested that the Keynote, if not sponsored by an organization, should be paid an honorarium by CRWAD. Council agreed, and in 2002 all sections had 45 min Keynotes. The scheduling of the Keynotes was staggered so that attendees could attend all the Keynotes if they desired. The Distinguished Veterinary Microbiologist became a Keynote speaker in one of the traditional microbiology sections in 2007. Mini-symposiums have also focused around the keynote speakers from time-to-time to explore the keynote's topic in greater depth.

Many organizations, referred to as CRWAD satellites, meet annually in conjunction with the annual CRWAD meeting. These organizations choose to meet with CRWAD so that their attendees, many of whom attend CRWAD, can save on travel time and expenses. Beginning in 1995 the CRWAD Executive Director and Administrative Assistant assisted the satellites in scheduling their meetings, and published their meeting dates and times in the CRWAD program. In addition to saving travel time and expenses, the satellites were often able to obtain meeting rooms under the CRWAD hotel contract at no charge. This relationship worked well as long as the satellite organization attendees were also attending CRWAD.

## The annual meeting – presentation format

The 15 September 1940 Announcement of the 21st Conference of Research Workers in Animal Diseases in North America states: “The policy of the Conference provides that contributors to the annual program shall be guided by the following: (1) Only new and unpublished data can be presented, (2) The paper must be presented in abstract and not read, (3) Presentation shall not exceed a time limit of 10 min. For the guidance of the program committee it is required that each title be accompanied by a brief abstract of the material to be presented. The abstract should be limited to 200 words.” The policy of short oral presentations has continued, with the long-standing time limit of 15 min. It is not known when the time limit, including questions, was extended to 15 min.

Over the years of CRWAD meetings, it is a tradition that all the sections keep the speakers strictly on time. Kitchen timers were used for decades to let the speakers know when the first 12 min had expired, and then again when the entire 15 min had passed. At the 1976 annual meeting, Drs Bob Ellis and Jack Schmitz were the section leaders for the Bacteriology section. After 12 min, a speaker was warned that he needed to conclude. The speaker assured Dr Ellis that he had ‘only a few more slides.’ Dr Ellis stopped the speaker when time expired. The same speaker had the next paper, so Dr Ellis introduced the next paper, and quickly reintroduced the same speaker. After 12 min, the speaker was told he had 3 min remaining, and again assured Dr Ellis he ‘only had a few more slides.’ The time expired, and the speaker continued to speak, so Dr Ellis ‘reeled him in’ with the wire that attached the mic to the speaker system, promptly and politely removed the mic from his neck, and introduced the next speaker, in a timely manner. The audience was appreciative that the section stayed on time. At the CRWAD meeting, many attendees have their schedules planned to move from section room to section room. If one or two sections are off schedule, presentations are missed and attendees do not gain the full benefit of the meeting.

The 26 November 1950, Council Meeting minutes state, “Although the increase in attendance at the annual conference has detracted from the original purpose of the conference which was to permit free and informal discussion of current investigations, the Council did not feel it desirable to limit the number of members.” It was agreed that no one who is a qualified investigator or who is directing research should be excluded from membership. This concept has carried through to the present time. The attendance is not limited to members and is extended to all who are presenting or who are membership candidates, and to many guests. In addition, during the 1952 meeting, it was agreed the meeting should be held at a hotel instead of at a University.

The President of CRWAD at the 1951 meeting, Dr W. L. Boyd, presented for discussion the desirability of having a 2-day meeting. The membership was “polled by mail to get the wishes of the members.” That proposition passed, and the oral presentations have been held on Monday and Tuesday since that meeting. Throughout the years, there have been full-day meetings or half-day meetings on Tuesday. The Business Meeting has been held traditionally just after the conclusion of the Tuesday morning scientific sessions. When there have been Tuesday afternoon sessions, it has been distinctly noted that the audience dwindled severely as the afternoon progressed. Accordingly, the last time there was a full day Tuesday meeting was 2001.

Another statement that was included in the 1 September 1952, Announcement for the 33rd annual meeting included additional information to the statement, ‘A lantern for the projection of slides of standard size will be available. A further comment which is very applicable even today stated a short poem written by Dr R. M. Hewitt, and published twice, AMA Bulletin 31:189–190 (December) 1936, and Pediatrics 7:145–149 (January) 1951,

“His audience yawned,

It squirmed and it sighed;

He constantly put

Too much on a slide.”

As we all can attest, this short poem holds true today, even after we have advanced from the lantern slides to the more common for decades 2 × 2 slides and now to PowerPoint electronic presentations. Even with seminars and on-line (web-based) courses specifically designed to enhance the format and ‘power’ of electronic presentations, it is common to see presentations that crowd too many lines per slide, and the font is so small that even those in the front of the room have difficulty reading the data.

## The annual meeting – sections

The earliest official note of more than one session was in 1955, when two sections were used to make room for more presentations. The two concurrent sessions were:

Section I: Bacteriology, immunology, parasitology, pathology
Section II: Physiology, biochemistry, nutrition, endocrinology

The sectioning of the meeting into several topical sessions has continued to the present. The most sections that have been designated are 10, and that was the number of individual sections for the 2014 meeting. In addition to the topical sections, there were several years where double sessions of certain sections were held in order to accommodate the number of abstracts for those sections. Even when there were two full days of the annual meeting, Monday and Tuesday, there were not enough 15-min time slots to accommodate all the abstracts. The year there were the most double sections was 1988, when two concurrent sessions each of the Bacteriology, Immunology, and Virology sections were held. It was also during this time that the annual meeting attendance peaked to 740, with an average annual attendance from 1990 to 1999 of 660. This arrangement allowed all the qualified abstracts to be presented, but met with dissatisfaction since even more scheduling conflicts arose when individuals wanted to go to presentations that were presented simultaneously.

The Physiology and Pharmacology section had received permission from Secretary-Treasurer Dr Erv Kohler to hold a small poster session in their meeting room in 1986. The 1990 annual meeting made poster presentation format available as an option for all sections. This reduced the scheduling conflicts, and has become a major mechanism of presentation at the annual meeting. The Poster Session I on Sunday evening had been coupled with the Researchers reception beginning in 1997. The reception allowed attendees to visit, view the posters, and enjoy the official opening of the annual meeting with hors d'oeuvres and refreshments. The idea for the reception was conveyed by a member to Administrative Assistant Suzy Squires at the 1996 meeting. Suzy and Bob Ellis discussed the mechanisms for establishing the reception, and Bob took the idea to the Council. The Council approved the concept if funding could be secured. The initial opening reception and all subsequent receptions have been generously funded by several corporate contributors. The Sponsorship Committee was appointed in 2008 through the encouragement of Administrative Assistant Suzy Squires. From previous years of contacting corporate sponsors, she was familiar with the process of requesting sponsorship from corporate sponsors, and believed that persons within the corporate community would be appropriate contacts. The Council agreed and the Sponsorship Committee was established in 2008. The Sponsorship Committee has worked throughout the years to maintain contacts within the corporate community and to ensure that the Researchers reception is adequately funded each year.

For many years CRWAD had been asked if we would allow commercial tabletop exhibits during our annual meeting. The Council agreed to allow tabletop exhibitors who had a definite application to animal disease research. Tabletop exhibitors were added for the 2007 CRWAD meeting and have continued since that year.

The Council had for decades noted that the graduate students were the future of the CRWAD. The first graduate student awards were presented at the 1986 meeting. At the 2014 Annual meeting, there were 23 student awards totaling $6425 in cash awards plus plaques, books, and association memberships. All awards were fully funded by organizations which had a vested interest in the CRWAD meeting. None of the funding was directly from CRWAD. There are also the AVEPM Schwabe Award, given annually to an outstanding veterinary epidemiologist, the Distinguished Veterinary Immunologist award given by the American Association for Veterinary Immunologists, and the Distinguished Veterinary Microbiologist award given by the American College of Veterinary Microbiologists. In 2012, the Student reception was initiated. All graduate students and post-doctoral fellows who were attending the annual meeting were invited to a reception prior to the first poster session. The purpose of the graduate student and post-doc reception was to give the young scientists an opportunity to have an informal mentoring session with the Dedicatee and Council. The discussion was lively and met the goals of the reception.

## The annual meeting – administrative challenges

As may be expected, there have been some unusual occurrences for the Secretary-Treasurers and Executive Directors of the CRWAD as follows:

Fortunately, Dr Kohler visited the Midland Hotel in early summer 1973, to check with the new management team. He judged the new Midland management to be incompetent so he made arrangements to hold the 1973–1975 meetings at the La Salle Hotel. Later that summer, the Midland had a fire which destroyed some of the meeting rooms. After 3 years of meetings at the La Salle, the hotel's owner died. Dr Kohler had asked the management to notify him if there was going to be a problem for their meeting. They called on a Monday in June and said they were closing that Friday. Dr Kohler went to Chicago and after visiting several hotels he selected the Pick Congress for future meetings.

After the 1991 meeting had concluded, Dr Ellis was going to count the cash with the Congress Hotel Finance Manager, and was told the Congress was not able to accept cash at that time, even when it was going to be used to pay the meeting expenses. That was a first! When does anyone not accept cash payment? Dr Ellis took the cash, about $5000, put it in his briefcase and walked a few blocks to a bank on Michigan Avenue. The bank was happy to issue a cashier's check, for a 10% fee of the total of the cashier's check. In view of the status of the finances, Dr Ellis elected to carry the cash back to the Congress Hotel, took it home with him and deposited the entire cash receipts from the meeting into the CRWAD account in Fort Collins. The trip home was without incident, and 100% of the cash was deposited into the CRWAD account.

The first year the annual meeting was held outside Chicago presented another challenge. During the summer of 2001, when Executive Director Dr Bob Ellis checked with the Millennium Hotel, he was told the Hotel had misplaced their contract, had changed Convention Managers, and said they did not have room for the meeting–all this in July! Bob insisted on talking to the General Manager, and was assured that they in fact had a meeting booked, and the hotel would stand by the contract. One satellite meeting was held at a nearby hotel, which the Millennium helped arrange.

The Congress Hotel was involved in a union strike in 2003 and 2004. The picketing did not impede people from attending their annual meeting but the chanting and noisemaking would continue loudly into the night. One CRWAD member opened his window at about 2 am, and threatened that he was going to start throwing the furniture out his hotel room window at the noisy strikers on the street below unless the noise stopped. The Executive Director was politely told by the hotel management the next morning that no hotel guests were to be throwing anything out the windows at the picketers. That was the only known confrontation between the picketers and the CRWAD hotel guests.

## The annual meeting – Proceedings

The printing of the Abstracts was initiated by Secretary-Treasurer Dr Erv Kohler for the 1973 meeting. At the Council's request, Dr Kohler invited Dr Arthur Freeman, Editor of the American Journal of Veterinary Research, to discuss possible publication of Abstracts in the Journal. Dr Freeman said it was not possible under their journal's structure thus ending that discussion. Beginning in 1973, Dr Kohler would send all the abstracts to Dr Howard Stowe of Michigan State University. Dr Stowe would receive the Abstracts as submitted by the presenters, and paste them up six to a page. After that was accomplished, the abstracts were taken to a printer to be printed into the Abstracts booklet. Dr Stowe would bring boxes of Abstracts booklets to the CRWAD meeting for distribution to the attendees. After Dr Stowe retired, Dr Ellis became the publisher of the Abstracts. The process was continued much as Dr Stowe had done it for a few years, then the printing of the Abstracts became digital. The name of the Abstracts was changed to CRWAD Proceedings in 1997. Beginning in 2000, Suzy Squires compiled the Program and Proceedings, and had them printed. Copies are available for purchase by individuals, and many are purchased each year by libraries worldwide.

The annual call for Abstracts continued to be mailed to all members, all student members, and those guests who had attended the past 2 years. The call for Abstracts was available online in 1998, and the mailing was discontinued after 2010. All calls for Abstracts were communicated through the CRWAD website. The Abstracts were received electronically as Microsoft Word documents beginning in 2000. Suzy Squires developed and managed a custom Microsoft Access database and form that digitally collected the authors’ titles, contact information, section preference, affiliation, and email address. She then compiled the Proceedings from the submitted Abstracts. Also in 2010 CRWAD started using the OASIS program. After 2010, both a Program-only booklet and a Program and Proceedings booklet were printed. The Program booklet was included in registration, the Program and Proceedings booklet was for purchase at an additional fee.

## Membership

The number of active members is not available for many of the early years of CRWAD. We do know that attendance, and thus membership, was strictly limited during the early years of the organization. The number of attendees in the early years was stated in the history section of this report. It is known that membership in 1943 was 164, in 1955 was 220, and had risen to 580 active members and 52 life members by 1977. The active membership had declined to 422 and the life members had increased to 105 by 2000. The active members had further declined to 263 and the life members had remained stable at 110 by 2014. The drop in membership had continued steadily from the late 1990s through 2014. Throughout the history of the CRWAD, 20–30 members were dropped annually for being 2 years in arrears in their dues, or by request. Each year, guests who attend and students who are graduating are encouraged to apply for membership.

CRWAD is truly an international organization as evidenced by the countries in which our members reside. Countries (14) represented in the CRWAD 2014 membership include Canada, China, Germany, India, Ireland, Japan, South Korea, Mexico, Switzerland, The Netherlands, Scotland, United Kingdom, USA, and West Indies. Countries (40) represented by guest attendees/registrations from 1995 to 2014 were Australia, Austria, Azerbaijan, Brazil, Canada, China, Czech Republic, Denmark, Egypt, Ethiopia, France, Georgia (Republic), Germany, India, Indonesia, Italy, Japan, Kazakhstan, Korea (Republic), Kosovo, Mexico, Mongolia, New Zealand, Nigeria, Norway, Pakistan, Philippines, Poland, Russian Federation, Slovakia, Sweden, Switzerland, Taiwan, Thailand, The Netherlands, Uganda, Ukraine, United Kingdom, USA, Vietnam, and West Indies.

A Membership directory was compiled, printed, and distributed to the membership in 1996 and 1997. The Membership directory was added to the website in 1997 and was no longer printed.

The Council developed the Student membership category in 1999. Students who register for the annual meeting are considered student members, and receive all the information that the active members receive. The student membership was created in order to recognize the significant contributions students and post-docs make to CRWAD, and to encourage them to become active members as they advance in their careers. There are currently several active members, some of whom have served as section leaders, who started as student members of CRWAD. President Lynn A. Joens in his 2007 President's Message in the Newsletter stated, “More importantly, the CRWAD meeting is the only International conference where student oral presentations remain the norm. This provides an International forum for students to display their state-of-the-art research and to hone their speaking skills.” In the same Newsletter, Executive Director Robert P. Ellis stated, “All categories of our membership (student, regular, and life) contribute to the sustainment and advancement of CRWAD as the premier international animal disease research organization.”

From the archives it was found that a newsletter was mailed to the membership 15 January 1955. It stated the “Origin and Purpose” of the Conference, defined membership, and gave details of the annual meeting. The newsletter noted, “Attendance at meetings is limited to members. There are a few exceptions. Certain guests may be invited by approval of the Council. This includes graduate students.” There probably were other newsletters sent to the members but information regarding additional newsletters was lacking until 1994. Dr Ellis commented in the 1994 Newsletter that “The 1994 Meeting will be the Seventy-Fifth Anniversary of the founding of the CRWAD. Our organization has moved from a small informal round-table discussion of animal disease research in the Midwestern U.S. to an international gathering of approximately 750 scientists. The 1996 Newsletter became the first of the annual newsletters that Bob and Suzy published, continuing through 2014. The 1996 Newsletter was mailed to all active members and life members. The newsletter has been posted annually on the CRWAD website since 1999.”

The first CRWAD website was constructed and managed by Suzy Squires in 1997. Suzy managed the website from inception through June 2015. At the time of the creation of the website, CRWAD did not have a logo. Bob Ellis suggested to Suzy that the logo should reflect the nickname by which many people call the CRWAD, ‘Crawdad’, a rough pronunciation of the acronym. Bob had a poster with many crayfish (crawdads) and they picked what they thought was the most colorful as the first CRWAD logo for use on the first website. In 1999, CABI Publishing designed and gifted to CRWAD the logo using the CRWAD letters looped by a ribbon denoting a DNA strand. In 2008 the CRWAD logo was further stylized by adding shadowed animals.

Communication with the membership and guests using an email list serve was initiated in 1998. Nearly all current communications are through the list serve and through our website. In addition to general communications regarding the annual meeting and the newsletter, there is a very active job listing site and other pertinent CRWAD information at the website.

## Animal Health Research Reviews

The founding fathers of the CRWAD were strictly against publishing any of the Proceedings from the annual meeting. A few attempts to do so met with stern rebukes and renewed reminders that the discussions at the meeting were not to be published. An excerpt from the 30 November 1943, minutes illustrates this debate as follows:

“A letter from a member suggesting that the Conference of Research Workers consider the possibility of sponsoring the publication of a new journal to provide for the publications of reports pertaining to research on food-producing and laboratory animals was discussed. After very careful consideration it was the unanimous opinion that such a venture is foreign to the objectives of the organization and could not properly become a part of its activities”.

In the words of Dr H. C. H. Kernkamp:

“A second small abscess showed up in 1946. This was when a member innocently, and I believe unmindfully, over stepped a tradition and hard-headed policy of the Conference by sending a report of the proceedings to a British veterinary publication” (H. C. H. Kernkamp, Appendix 2)

The Council Minutes for 2 December 1946, compiled by Secretary Dr William H. Feldman, stated the resolution of the above incident:

“The publication in the September, 1946 issue of the Veterinary Journal (British) of a rather comprehensive but unauthorized report of the proceedings of the Conference of Research Workers in Animal Diseases for 1945 was the subject of careful consideration by the Council. The responsibility for this unfortunate incident was definitely determined and a statement expressing regret and apology has been received. The Council hopes that this unfortunate violation of our unwritten rule that information divulged at our scientific sessions should not be publicized, will have a deterrent effect on members and guests who may have reportorial aspirations.”

The discussion of even printing the abstracts was rejected at the business meeting on 1 December 1953, wherein it was stated that the members “voted on and rejected the printing of abstracts in the program because the printing of the abstracts would make public the proceedings of the Conference.”

We know that the Council and the members at the business meeting in later years changed their minds, and Abstracts were published beginning in 1973 and continuing through the present.

Prior to the Council meeting on 7 November 1998, Philip Edge from the Center for Agriculture and Biosciences International (CABI) Press contacted Executive Director Bob Ellis with a proposal for an International Animal Health and Disease Review Journal. After the initial contact, Bob invited Mr Edge to attend the Council meeting and present his proposal. Mr Edge proposed a review journal to the Council and his proposal was met with positive enthusiasm. The 1998 Council minutes state,

“CABI had searched for current Animal Health Review journals and found that none existed. The journal would possibly be called Animal Health Reviews, and the entire costs and marketing would be borne by CABI. CRWAD would be a source of editors and authors. Dr. Ellis would likely be the Editor-in-Chief, with others on the editorial board coming from the Council and membership. The editors would not be exclusively from CRWAD, nor would the authors. The breadth and depth of animal health and disease knowledge among the members of CRWAD would probably mean that most of the editors would be CRWAD members, and most of the articles would be contributed by CRWAD members. Another advantage for our members would be a reduced subscription rate for the review journal. Initially, it is planned for the journal to be launched in 1999, with two issues for the first volume. Each issue would have approximately 6 articles. The articles would likely cover a spectrum of animal health worldwide. In addition, CABI asked permission to send a questionnaire to our members in order to gain more insight into the members’ support and suggestions regarding the proposed journal.”

Permission was granted, and Dr Ellis was authorized to work with CABI to formulate and distribute the questionnaire. The Council and members approved that proposal, and the first issue of Animal Health Research Reviews (AHRR) was published in June 2000. The AHRR is an international journal which has been published each June and December since 2000. Dr Robert P. Ellis (2000–2002) was the first Editor-in-Chief, followed by Dr Carlton Gyles (2002–2012). In 2006, Cambridge University Press became the publisher of the AHRR. Dr Roger W. (Bill) Stich is the current Editor-in-Chief (2013-present).

## Supplementary materials and methods

The supplementary material for this article can be found at http://www.journals.cambridge.org/AHR

